# Heat stress and corn quality affect amino acid digestibility in broiler chickens

**DOI:** 10.1016/j.psj.2026.107333

**Published:** 2026-06-23

**Authors:** T.J. Alabi, S.F. Olayiwola, M.D. Lindemann, A.J. Pescatore, J.P. Jacob, M.J. Ford, S.A. Adedokun

**Affiliations:** Department of Animal and Food Sciences, University of Kentucky, Lexington, KY, USA

**Keywords:** Broiler, Corn fines, Endogenous amino acid, Heat stress, Standardized ileal digestibility

## Abstract

Corn constitutes over 50% of broiler diets and contributes measurable amino acids (**AA**), but several factors could affect its eventual net AA supply. Heat stress (**HS**) reduces feed intake and may compromise intestinal integrity and increase endogenous AA losses. A total of 384 one-day-old male Cobb by-product breeder chicks were allocated to 6 treatments (3 diets × 2 environmental temperatures) using a randomized complete block design with 8 replicate cages of 8 birds/cage. All birds received a standard corn-soybean meal-based starter diet until experimental diets were introduced. Experimental diets included a nitrogen-free diet (**NFD**) and semi-purified diets containing either regular corn or mycotoxin-contaminated corn fines. The NFD was fed for 4 d and the semi-purified diets for 6 d prior to ileal digesta collection on d 22. Data were analyzed using PROC GLM of SAS, with endogenous ileal AA data analyzed using the one-way ANOVA, while apparent ileal amino acid digestibility (**AIAAD**) and standardized ileal amino acid digestibility (**SIAAD**) data were analyzed as a 2 × 2 factorial arrangement of treatments. Cyclic HS increased (*P* < 0.05) endogenous ileal AA losses for nitrogen, and all AAs, with total AA losses increased by 36.5%. Mycotoxin-contaminated corn fines decreased (*P* < 0.001) AIAAD and SIAAD across all AAs compared to regular corn. There were diet × temperature interactions (*P* < 0.05) for five AAs (lysine, methionine, valine, aspartate, and glycine), where HS decreased (*P* < 0.05) AIAAD in birds fed mycotoxin-contaminated corn fines. For SIAAD, a main effect of temperature (*P* < 0.05) occurred, with HS birds exhibiting higher values than thermoneutral (**TN**) birds for most AAs (except tryptophan, cysteine, and proline). Nevertheless, the combination of corn fines and HS resulted in the lowest (*P* < 0.05) SIAAD for arginine, isoleucine, lysine, phenylalanine, valine, aspartic acid, and glycine. Birds under HS fed regular corn had the highest (*P* < 0.05) SIAAD for these AAs. These results demonstrate that SIAAD coefficients derived under TN conditions are not directly additive under HS.

## Introduction

In recent years, heat stress **(HS)** has been identified as a major global environmental stressor that adversely affects poultry health and performance, especially in hot-climate regions and during the summer months in temperate regions. Birds are vulnerable to HS due to limited heat-dissipating capacity from feathering and lack of sweat glands (Teyssier et al., 2023). As homeothermic animals, they require a narrow temperature range for optimal physiological function (Olayiwola and Adedokun, 2025). When exposed to high temperatures, they undergo several behavioral and physiological adaptations, including reduced feed intake **(FI)** and compromised intestinal integrity (Rostagno, 2020; Wang et al., 2018). This occurs partly because HS redistributes blood flow away from the splanchnic region (Al-Beltagi et al., 2025), inducing oxidative stress (Brugaletta et al., 2022; Rostagno, 2020) and damaging the intestinal mucosa (Nanto-Hara et al., 2020), collectively impairing the digestion and absorption of nutrients, including amino acids **(AA)** (Wallis and Balnave, 1984). Although the negative effects of HS on feed efficiency and growth in poultry production are well documented, studies exploring its impact on AA digestibility remain limited.

Profitability in poultry production depends on effective feed management and the provision of nutritionally adequate, high-quality diets that support bird health and performance. Corn and soybean meal **(SBM)** constitute the primary ingredients in commercial poultry rations, accounting for over 90% of conventional corn-SBM-based diet formulations, with corn alone often exceeding 50% of total diet inclusion. Although corn is primarily included as an energy source (Cowieson, 2005), it also contributes measurable amounts of crude protein **(CP)** and AAs (Zuber and Rodehutscord, 2017), which must be accounted for in diet formulation. Cowieson (2005) reported that the nutritional value and quality of corn in poultry diets can differ due to factors such as cultivar, drying temperature, growing conditions, starch content, protein structure, fiber composition, and the presence of anti-nutritional factors **(ANF).** Of particular concern is the use of mycotoxin-contaminated corn fines, a fragment derived from corn grain screening, which is often concentrated with mycotoxins such as fumonisins and aflatoxins (Schaarschmidt and Fauhl-Hassek, 2021). These mycotoxins are known to impair intestinal barrier function and nutrient absorption (Chen et al., 2016). Variations in corn quality can influence AA digestibility and absorption in broilers. Key essential AAs such as lysine, methionine, threonine, valine, and isoleucine show minimal variation across corn sources (Vargas et al., 2023).

Apparent ileal amino acid digestibility (**AIAAD**) and standardized ileal amino acid digestibility (**SIAAD**) are key metrics for evaluating ingredient quality. Several studies have reported high AIAAD and SIAAD values for corn under standard conditions (Adedokun et al., 2015; Lagos and Stein, 2017; Rochell et al., 2016). However, the AIAAD value is influenced by basal ileal endogenous amino acid losses (**BIEAAL**), which include proteins from digestive secretions, mucins, and sloughed enterocytes (Ravindran and Bryden, 1999). These endogenous losses are not static; they can be substantially increased by dietary factors such as fiber or mycotoxins, and by physiological stressors, including disease and HS (Adedokun et al., 2007a; Cowieson et al., 2004). Therefore, under challenging conditions, AIAAD can significantly underestimate the true digestible AA supply because it conflates undigested dietary AAs with increased endogenous losses. In contrast, SIAAD corrects for basal endogenous losses, providing a more accurate, consistent, and additive estimate of truly digestible AAs, making it the preferred metric for feed formulation under varying physiological states (Lemme et al., 2004; Ravindran, 2016). Consequently, modern poultry feed formulation has shifted toward utilizing the SIAAD systems to enhance formulation precision, improve nitrogen utilization, and reduce environmental impact (Alabi and Adedokun, 2025).

Despite the advantage of SIAAD for estimating true AA digestibility under varying conditions, a critical gap remains in applying this principle to real-world poultry production stressors. While the separate effects of HS on gut physiology and of mycotoxin-contaminated corn fines on bird health and general performance are documented (Grenier and Applegate, 2013; Lara and Rostagno, 2013), their combined impact on SIAAD remains unquantified. It is unknown whether the increase in endogenous losses induced by HS exacerbates the digestive limitations of low-quality corn, leading to a disproportionate reduction in digestible AA supply. Quantifying this interaction is essential for formulating diets that can maintain AA adequacy under the realistic, combined pressures of high environmental temperatures and variable feedstuff quality.

Therefore, the objective of this study was to evaluate the individual and interactive effects of cyclic HS and corn quality (regular corn vs. mycotoxin-contaminated corn fines) on BIEAAL, AIAAD, and SIAAD in 22-day-old broiler chickens. We hypothesized that HS would increase BIEAAL, and that mycotoxin-contaminated corn fines would reduce AIAAD and SIAAD, with the combination of both stressors leading to a detrimental effect on SIAAD, particularly for critical AA in broiler chickens.

## Materials and Methods

### Animal ethics statement

All experimental procedures and management of birds were approved by the Institutional Animal Care and Use Committee of the University of Kentucky.

### Experimental Animals, housing, design, and diet

A total of 384 birds (1-day-old male Cobb by-product breeder chicks) were obtained from a commercial hatchery. The birds were raised in battery cages (0.61 × 0.51 × 0.36) m^3^ (Model SG12-24 × 24 × 18-PVC-U, Alternative Design Manufacturing, Siloam Springs, AR, USA) located in two environmentally controlled rooms with different temperatures - thermoneutral **(TN)** and HS**,** and 22 h of light and 2 h of darkness. The study was conducted as a 3 (diets) x 2 (room temperatures) factorial arrangement of treatments in a randomized complete block design with location in the room as the blocking factor. To check for the room effect, two rooms were used for each of the two levels of temperature. At the start of the trial, all birds were raised at 32 °C for the first 9 d. During subsequent wks (wk 2 to 3), the room temperature of the birds on HS was increased and maintained at 34.3 °C for 6 h/d (15:00 to 21:00 GMT) until the end of the study, while the room temperature for the birds under the TN temperature was maintained at 29.4 °C (wk 2) and 26.5 °C (wk 3). At the end of the 6 h of exposure to HS, the room temperature was reverted to that of the birds exposed to the TN temperature (29.4 and 26.5 °C, respectively) from 21:00 until 15:00 GMT the following day. It took about 30 minutes for the room temperature to revert to the TN level, while it took less than 30 minutes for the room to reach the HS level. This temperature regimen was repeated until the end of the study (d 22). Each diet was replicated four times within each room with 8 birds/replicate cage, resulting in 8 replicate cages/dietary treatment (room effect was not significant for the two room temperatures).

Birds were fed a corn-SBM-based diet (Table 1) that met or exceeded their nutrient and energy requirements (Cobb-Vantress, 2015) until they were fed the experimental diets. The three experimental diets formulated for this study were a nitrogen-free diet (**NFD**), a semi-purified diet containing regular corn, and a semi-purified diet containing field mycotoxin-contaminated corn fines (Table 2). The semi-purified diets containing either the regular corn or mycotoxin-contaminated corn fines were fed to the birds in the semi-purified diet group from d 16-22 (6 days), while the NFD was fed from d 18-22 (4 days) for the NFD group. Titanium dioxide was added to the experimental diets at 0.5% as an indigestible marker to estimate nutrient digestibility. The corn (regular corn) used in this study was cultivated locally and screened to produce corn fines as described by Paczosa et al. (2025). The mycotoxin analysis of the regular corn and corn fines showed deoxynivalenol (0.74 vs. 11.22 ppm), fumonisins (1.07 vs. 35.85 ppm), and Zearalenone (<0.25 vs. 1.22 ppm) to be the prevailing mycotoxins in the corn fines. The analyzed AA values for both corn samples are presented in Table 3.

**Table 1:** Feed ingredients composition and calculated nutrient content of the broiler starter diet (on as-fed basis)^1^.

Ingredients, g/kg	Starter diet
Corn	602.4
Soybean meal (47% CP)	330
Soy oil	28.0
L-Lysine HCl	2.6
DL-Methionine	3.4
L-Threonine	0.9
Salt (NaCl)	4.0
Limestone	9.85
Dicalcium phosphate	16.6
Vitamin-mineral premix^2^	2.3
Total	1,000
Calculated nutrients and energy, g/kg	
Crude protein	210
MEn, kcal/kg	3,101
Calcium	9.1
Phosphorus	7.0
Non-phytate phosphorus	4.5
Amino acid, g/kg	
Lys	13.32
Met	6.32
Met + Cys	9.78
Thr	8.64
Trp	2.54

1 Starter diet was fed from d 0-16 for the semi-purified groups and d 0-18 for the NFD group.

2 Provided the following quantities of vitamins and micro minerals per kilogram of complete diet: iron from iron sulfate, 32 mg; copper from copper sulfate, 8 mg; manganese from manganese oxide, 51 mg; zinc from zinc oxide, 60 mg; iodine (EDDI), 1.48 mg; selenium as sodium selenite, 0.24 mg; vitamin A (retinyl acetate), 8,820 IU; vitamin D_3_ (cholecalciferol), 2,822 IU; vitamin E (dl-α-tocopheryl acetate), 26 IU; vitamin K activity, 0.73 mg; thiamin, 1.76 mg; riboflavin, 6.17 mg; pantothenic acid, 14 mg; niacin, 44 mg; pyridoxine, 4 mg; folic acid, 0.88 mg; biotin, 0.18 mg; vitamin B_12_, 0.02 mg; choline, 383 mg.

**Table 2:** Feed ingredients composition of the experimental diets (on as-fed basis).

		Semi-purified diet^2^	
Diet Type	Nitrogen-free diet^1^	Regular corn	Corn fines
Ingredient g/kg			
Corn starch	199.8	100.0	100.0
Dextrose	640	131.7	131.7
Solkafloc	50	30	30
Corn-regular	0.0	650	0.0
Mycotoxin-contaminated corn^3^	0.0	0.0	650
Soybean oil	50	50	50
Vitamin-mineral premix^4^	2.3	2.3	2.3
Dicalcium phosphate	24.1	16.5	16.5
NaHCO_3_	6.0	5.3	5.3
KCl	5.0	0	0.0
MgO	2.0	0	0.0
Limestone	7.8	9.2	9.2
K_2_CO_3_	8	0.0	0.0
Titanium dioxide^5^	5.0	5.0	5.0
Total	1,000	1,000	1,000

1 Nitrogen-free diet was fed from d 18-22.

2 Semi-purified diets were fed from d 16-22.

3 Contained deoxynivalenol, 11.22 ppm; fumonisins, 35.85 ppm; zearalenone, 1.22 ppm.

4 Provided the following quantities of vitamins and micro minerals per kilogram of complete diet: iron from iron sulfate, 32 mg; copper from copper sulfate, 8 mg; manganese from manganese oxide, 51 mg; zinc from zinc oxide, 60 mg; iodine (EDDI), 1.48 mg; selenium as sodium selenite, 0.24 mg; vitamin A (retinyl acetate), 8,820 IU; vitamin D_3_ (cholecalciferol), 2,822 IU; vitamin E (dl-α-tocopheryl acetate), 26 IU; vitamin K activity, 0.73 mg; thiamin, 1.76 mg; riboflavin, 6.17 mg; pantothenic acid, 14 mg; niacin, 44 mg; pyridoxine, 4 mg; folic acid, 0.88 mg; biotin, 0.18 mg; vitamin B_12_, 0.02 mg; choline, 383 mg.

5 Added to the diets as an indigestible marker for digestibility calculations.

**Table 3:** Analyzed nitrogen and amino acid composition of the regular and mycotoxin-contaminated corn samples.

	Regular corn	Corn fines
Nitrogen, g/kg	14.0	14.7
*Essential amino acid, g/kg*		
Arginine	4.40	3.50
Histidine	2.90	2.60
Isoleucine	3.60	3.45
Leucine	10.85	10.20
Lysine	3.40	2.95
Methionine	1.85	1.45
Phenylalanine	4.55	4.35
Threonine	3.15	3.10
Tryptophan	0.65	0.64
Valine	4.50	4.25
*Nonessential amino acid, g/kg*		
Alanine	6.60	6.25
Aspartic acid	6.35	6.55
Cysteine	2.05	1.80
Glutamic acid	17.10	15.35
Glycine	3.50	3.90
Proline	7.30	6.35
Serine	3.85	3.55
Tyrosine	3.30	2.95
Total amino acid	91.60	85.34

### Sample collection and Laboratory analysis

On d 22, birds were euthanized by argon asphyxiation, and digesta from the entire ileum from all birds within each cage were flushed into a clean plastic container, pooled, and stored at −20 °C until they were freeze-dried. Dried ileal digesta samples were ground using a coffee grinder prior to chemical analysis. Diets, regular corn, and mycotoxin-contaminated corn fines samples were ground with a Wiley Mill Laboratory Standard grinder (Model No. 3, Arthur H. Thomas Co., Philadelphia, PA, USA) fitted with a 0.5 mm screen.

The DM contents of the diets and ileal digesta were determined by drying the samples at 105°C for 24 h in a forced-air drying oven (VWR Gr Con 3.7CF, Radnor, PA; method 934.0) (AOAC, 2006). The gross energy of the diets and ileal digesta was determined using a Parr adiabatic bomb calorimeter (model 6200, Parr Instruments, Moline, IL, USA) with benzoic acid as the calibration standard. Nitrogen was determined by combustion method using a LECO analyzer (Model FP2000, LECO Corp., St. Joseph, MI; method 990.03) (AOAC, 2000), with ethylene diamine tetraacetic acid (**EDTA**) as the internal standard. Titanium concentration (in diets and ileal digesta) and AA contents of the corn and mycotoxin-contaminated corn fines samples, diets, and ileal digesta were determined at the University of Missouri Experiment Station Chemical Laboratory (Columbia, MO). Amino acid concentrations in diets and ileal digesta were analyzed using method 982.30 E (a, b, c); (AOAC, 2006) as described by Adedokun et al. (2012). Titanium concentrations in diets and ileal digesta were determined by flame atomic absorption spectroscopy after the samples were digested using concentrated sulfuric acid and processed as described by Myers et al. (2004).

### Nutrient digestibility calculations

The BIEAAL were calculated using the index method by Adedokun et al. (2017):

$$\text{BIEAAL}\;\left(\text{mg}/\text{kg}\;\text{of}\;\text{DMI}\right)=\left(\frac{Ti_{i}}{Ti_{o}}\right)\times \left(N_{o}\right)$$

where Ti_i_ and Ti_o_ represent the concentration of titanium (g/kg DM) in the diet and ileal digesta, respectively, while N_o_ represents the N or AA concentration in the ileal digesta.

The apparent ileal digestibility (**AID**) of N and AA was calculated using the index method (Adejumo et al., 2021).

$$\text{Apparent}\;\text{ileal}\;\text{digestibility}\;\left(\%\right)=\left[1-\left(\frac{Ti_{i}}{Ti_{o}}\right)\times \left(\frac{N_{o}}{N_{i}}\right)\right]\times 100$$

where Ti_i_ represents the concentration of titanium in the diet in g/kg of DM; Ti_o_ represents the concentration of titanium in the ileal digesta in g/kg of DM output; N_o_ represents the concentration of N or AA in ileal digesta in mg/kg of DM; N_i_ represents the concentration of N or AA in diets in mg/kg of DM. The BIEAAL values obtained for birds that were fed the NFD within each of the sampling cages were used to standardize the apparent ileal N and AA digestibility values obtained from birds that were fed the respective semi-purified diets using the following formula (Adedokun et al., 2017):

SIAAD,(%)=AID(%)+[100×(BIEAALing/kgofDMI)÷(AAcontentdieting/kgofDM)]

where SIAAD = standardized ileal amino acid digestibility; AID = apparent ileal N or AA digestibility; BIEAAL = basal ileal endogenous amino acid digestibility.

The potential influence of room effect on the data was tested using the t-test, which was not significant (*P* > 0.05). Afterward, data from each of the two rooms (within each temperature) treatment were pooled, leading to 8 replicate cages per dietary treatment for statistical analysis.

### Statistical analysis

Prior to conducting statistical analysis, all outliers (data outside the mean ± 3 standard deviation) were removed from the data set. Data were analyzed as a randomized complete block design using the PROC GLM procedure of SAS v. 9.4 (SAS Institute Inc., Cary, NC). Data for BIEAAL were analyzed using a one-way ANOVA, while data for AIAAD and SIAAD were analyzed as a factorial arrangement of treatments with 2 temperature levels (TN vs. HS) and 2 semi-purified diets (the regular corn or mycotoxin-contaminated corn fines). The significance level was set at *P* < 0.05, and 0.05 < *P* ≤ 0.10 was considered a statistical tendency. Where necessary, mean separation was performed using Tukey's Honestly Significant Difference test.

## Results

The analyzed nitrogen and AA contents of the two corn samples are reported in Table 3. With the exception of aspartic acid and glycine, the concentration of AAs in the mycotoxin-contaminated corn fines were numerically lower than that of the regular corn, and this is also reflected in the composition of the total amino acids (**TAA**). The TAA content was 6.8% higher in the regular corn than in the mycotoxin-contaminated corn fines. The average values for the essential amino acids (**EAAs**) were 3.98 and 3.65 g/kg for regular corn and mycotoxin-contaminated corn fines, respectively, while the corresponding values for the nonessential amino acids (**NEAAs**) were 6.26 and 5.84 g/kg. Contrary to the AA contents, the nitrogen concentration of the mycotoxin-contaminated corn fines was 5% higher than that of the regular corn.

The chemical analysis of the experimental diets (Table 4) shows that the semi-purified corn fine diet had a higher nitrogen content than the semi-purified regular corn diet. This is similar to the nitrogen content of the regular corn and corn fines as reported in Table 3. The NFD contained very small amounts of nitrogen and TAA. The semi-purified mycotoxin-containing corn fines diet had higher nitrogen and TAA values than the regular corn diet.

**Table 4:** Analyzed nitrogen and amino acid composition of the experimental diets (on as-fed basis).

		Semi-purified diet	
	Nitrogen-free diet	Regular corn	Corn fines
Nitrogen, g/kg	0.50	8.43	9.51
*Essential amino acid, g/kg*			
Arginine	0.00	2.35	2.30
Histidine	0.10	1.90	1.85
Isoleucine	0.00	2.05	2.20
Leucine	0.10	6.55	6.50
Lysine	0.10	1.95	2.00
Methionine	0.00	1.00	0.80
Phenylalanine	0.00	2.65	2.80
Threonine	0.00	1.80	2.00
Tryptophan	<0.20	0.52	0.58
Valine	0.00	2.55	2.65
*Nonessential amino acid, g/kg*			
Alanine	0.00	3.95	4.00
Aspartic Acid	0.00	3.40	4.00
Cysteine	0.00	1.15	1.10
Glutamic Acid	0.10	9.75	9.60
Glycine	0.00	2.00	2.35
Proline	0.05	4.35	4.05
Serine	0.00	2.25	2.35
Tyrosine	0.00	2.05	2.10
Total amino acid	1.70	54.37	55.58

The environmental temperature did not affect (*P* = 0.409) the daily FI of the broiler chickens fed the NFD. The effects of environmental temperature and diet quality on FI are presented in Table 5. Main effect of temperature was observed (*P* = 0.019), with HS reducing average daily feed intake (**ADFI**) by approximately 5.5% compared with birds in the TN group. In contrast, diet quality did not influence FI (*P* = 0.637). Furthermore, there was no interaction between temperature and diet quality (*P* = 0.875).

**Table 5:** Effect of environmental temperature and diet quality on the feed intake of broiler chickens.

Diet quality	Temperature	Average feed intake/d, g
	Thermoneutral	71.29
	Heat Stress	67.36
		
Regular Corn		68.95
Contaminated Fines		69.70
		
Regular Corn	Thermoneutral	70.79
Contaminated Fines	Thermoneutral	71.79
Regular Corn	Heat Stress	67.11
Contaminated Fines	Heat Stress	67.61
	SEM:	1.112
	P-values	
	Temperature	0.019
	Diet quality	0.637
	Temperature × diet quality	0.875

Cyclic HS increased the nitrogen and IEAAL in the broiler chickens fed the NFD (Table 6). The total nitrogen loss was higher (*P* = 0.003) in heat-stressed birds compared to those in the TN group. Similarly, the TAA losses increased by 36.5% under HS conditions (*P* < 0.001). Among the EAAs, the most substantial increases were observed for methionine, lysine, arginine, and valine. For NEAAs, alanine, glutamic acid, and aspartic acid exhibited the greatest increase in (*P* < 0.001) endogenous flow under HS.

**Table 6:** Effect of cyclic heat stress on nitrogen and ileal endogenous amino acids loss in birds fed nitrogen-free diet.

	Temperature^1^			Probability
	Thermoneutral	Heat stress	SD^2^	
Nitrogen	1,131	1,487	138	0.003
*Essential amino acid, mg/kg DMI*				
Arginine	182	298	33.2	<.001
Histidine	122	179	21.2	<.001
Isoleucine	222	326	31.3	<.001
Leucine	315	479	48.0	<.001
Lysine	235	403	46.1	<.001
Methionine	42	79	9.9	<.001
Phenylalanine	199	295	30.0	<.001
Threonine	410	538	34.4	<.001
Tryptophan	45	61	5.4	0.01
Valine	250	386	36.6	<.001
*Nonessential amino acid, mg/kg DMI*				
Alanine	209	322	28.6	<.001
Aspartic acid	421	642	60.5	<.001
Cysteine	168	210	21.6	0.002
Glutamic acid	467	745	81.0	<.001
Glycine	252	353	31.9	<.001
Proline	300	397	31.0	<.001
Serine	323	411	32.3	<.001
Tyrosine	182	246	22.7	0.001
Total amino acid	5,547	7,573		

1 Number of replicates for birds raised under thermoneutral and heat stress conditions were 8 and 7, respectively.

2 SD = standard deviation.

The effect of temperature and corn quality on AIAAD is presented in Table 7. There was a main effect of diet quality, with regular corn exhibiting higher (*P* < 0.001) apparent ileal digestibility for nitrogen and AAs compared to mycotoxin-contaminated corn fines. On average, the AIAAD of TAA was 79.9% for regular corn and 64.4% for mycotoxin-contaminated corn fines. While the main effect of temperature was not significant for most response variables, interaction between diet and temperature was observed for some EAAs, including lysine (*P* = 0.026), methionine (*P* = 0.030), arginine (*P* = 0.025), and valine (P = 0.048). For these AAs, HS further reduced digestibility in birds fed the mycotoxin-contaminated corn fines diet but had no detrimental effect in birds fed the regular corn diet. For NEAAs, significant interactions were observed for aspartic acid and glycine with the combination of HS and mycotoxin-contaminated corn fines, resulting in lower (*P* < 0.05) AIAAD. Nitrogen and 5 AAs (isoleucine, leucine, phenylalanine, alanine, and glutamic acid) showed a tendency for an interaction between HS and corn quality, with the lowest (*P* < 0.10) AA digestibility for mycotoxin-contaminated corn fines in HS.

**Table 7:** Simple and main effect of room temperature and corn quality on apparent ileal amino acid digestibility in 22-day-old broiler chickens (%)^1^.

	Diet		Temperature^2^		Thermoneutral		Heat stress			Probability		
	Regular corn	Corn fines	TN	HS	Regular corn	Corn Fines	Regular corn	Corn fines	SD^3^	Diet	Temp^4^	Diet × Temp
Dry matter	78.0	70.6	74.6	74.0	78.0	71.3	78.0	70.0	2.23	<.001	0.453	0.453
Nitrogen	73.9	56.9	65.4	65.4	73.0	57.9	74.7	56.0	2.70	<.001	0.937	0.086
*Essential amino acid*												
Arginine	83.4	67.0	75.9	74.4	83.0^a^	68.8^b^	83.7 ^a^	65.1^b^	2.43	<.001	0.122	0.025
Histidine	81.3	64.6	72.9	73.0	80.7	65.1	81.9	64.1	2.56	<.001	0.934	0.275
Isoleucine	78.4	63.8	71.4	70.8	77.7	65.1	79.1	62.4	2.88	<.001	0.559	0.065
Leucine	87.9	76.2	82.1	82.0	87.3	77.0	88.6	75.4	2.01	<.001	0.850	0.067
Lysine	71.3	49.3	61.4	59.2	70.9^a^	51.9^b^	71.7^a^	46.7^c^	3.42	<.001	0.103	0.026
Methionine	87.4	69.2	79.1	77.4	87.0^a^	71.3^b^	87.7^a^	67.1^c^	2.81	<.001	0.118	0.030
Phenylalanine	83.6	70.9	77.5	77.1	83.0	72.0	84.3	69.9	2.28	<.001	0.618	0.054
Threonine	57.9	37.4	47.3	48.0	56.7	37.9	59.1	36.9	4.24	<.001	0.659	0.285
Tryptophan	83.9	75.7	79.8	79.8	83.7	75.9	84.0	75.6	3.36	<.001	0.994	0.816
Valine	77.9	59.6	69.6	67.9	77.6^a^	61.6^b^	78.1^a^	57.6^b^	2.99	<.001	0.131	0.048
*Nonessential amino acid*												
Alanine	85.9	72.1	79.1	78.8	85.3	73.0	86.4	71.1	2.30	<.001	0.680	0.092
Aspartic acid	72.4	57.6	65.3	64.7	71.3^a^	59.3^b^	73.4^a^	56.0^b^	3.31	<.001	0.657	0.038
Cysteine	69.1	38.6	54.6	53.1	69.0	40.1	69.3	37.0	4.20	<.001	0.372	0.285
Glutamic acid	88.1	75.6	82.0	81.6	87.6	76.5	88.6	74.7	1.93	<.001	0.589	0.063
Glycine	67.7	46.5	57.4	56.9	66.6^a^	48.1^b^	68.9^a^	44.9^b^	3.31	<.001	0.694	0.033
Proline	82.4	64.6	73.6	73.5	81.9	65.3	83.0	64.0	2.50	<.001	0.955	0.210
Serine	75.0	58.5	65.7	67.8	73.6	57.8	76.4	59.1	3.17	<.001	0.084	0.541
Tyrosine	82.9	72.5	77.4	78.1	82.0	72.8	83.9	72.3	2.15	<.001	0.393	0.160
Total amino acid	79.9	64.4	72.2	72.1	79.1	65.3	80.6	63.6	2.53	<.001	0.895	0.111

1 Number of replicates were 7 for all the simple effect groups except for the corn fines and thermoneutral temperature with 8 replicates.

2 TN = thermoneutral; HS = heat stress.

3 SD = standard deviation.

4 Temp = temperature.

The effects of corn quality and temperature on nitrogen and SIAAD are reported in Table 8. Diet quality affected SIAAD (*P* < 0.001), with regular corn consistently having higher (P < 0.001) SIAAD values than mycotoxin-contaminated corn fines for all AAs and nitrogen. The SIAAD for TAAs was 90.6% for regular corn and 75.0% for mycotoxin-contaminated corn fines. A main effect of temperature (*P* = 0.015) was observed for TAA, with heat-stressed birds exhibiting higher SIAAD values than those in the TN environment (except for tryptophan, cysteine, and proline). Significant interactions between diet and temperature were observed for arginine (P = 0.024), isoleucine (P = 0.041), lysine (*P* = 0.025), phenylalanine (*P* < 0.039), valine (*P* = 0.022), aspartic acid (*P* < 0.018), and glycine (*P* < 0.022).

**Table 8:** Simple and main effects of room temperature and corn quality on standardized ileal amino acid digestibility in 22-day-old broiler chickens (%)^1^.

Corn	Regular corn	Corn fines	Temperature^2^			Thermoneutral		Heat stress			Probability		
Temperature^2^			TN	HS		Regular corn	Corn fines	Regular corn	Corn fines	SD^3^	Diet	Temp^4^	Diet × Temp
Nitrogen	87.6	69.4	77.0	80.0		85.1	68.8	90.0	70.0	2.76	<.001	0.006	0.091
*Essential amino acid*													
Arginine	92.4	76.3	83.1	85.6		90.0^b^	76.3^c^	94.7^a^	76.4^c^	2.54	<.001	0.016	0.024
Histidine	88.2	71.9	78.8	81.3		86.4	71.1	90.0	72.6	2.50	<.001	0.012	0.264
Isoleucine	90.4	75.0	81.0	84.3		87.6^b^	74.5^c^	93.1^a^	75.4^c^	2.90	<.001	0.006	0.041
Leucine	93.4	82.0	86.7	88.6		91.7	81.6	95.0	82.3	1.96	<.001	0.012	0.085
Lysine	85.6	63.6	72.1	77.1		81.6^b^	62.6^c^	89.6^a^	64.6^c^	3.41	<.001	0.001	0.025
Methionine	93.1	76.4	83.6	85.9		91.0	76.1	95.1	76.6	2.94	<.001	0.046	0.103
Phenylalanine	91.9	78.8	84.2	86.5		89.9^b^	78.5^c^	94.0^a^	79.0^c^	2.25	<.001	0.010	0.039
Threonine	81.7	58.9	67.6	72.9		78.1	57.1	85.3	60.6	4.16	<.001	0.002	0.244
Tryptophan	93.1	84.1	87.6	89.6		91.9	83.3	94.3	85.0	3.25	<.001	0.096	0.781
Valine	88.8	70.4	78.2	81.0		86.0^b^	70.4^c^	91.6^a^	70.4^c^	3.04	<.001	0.020	0.022
*Nonessential amino acid*													
Alanine	91.8	78.0	84.0	85.8		90.1	77.9	93.4	78.1	2.30	<.001	0.048	0.090
Aspartic acid	86.4	69.4	75.7	80.1		82.6^b^	68.8^c^	90.1^a^	70.0^c^	3.36	<.001	0.002	0.018
Cysteine	83.9	54.2	68.1	70.0		82.1	54.0	85.6	54.4	4.26	<.001	0.235	0.353
Glutamic acid	93.4	81.2	86.4	88.3		91.7	81.0	95.1	81.4	2.08	<.001	0.020	0.064
Glycine	80.9	58.0	67.7	71.1		77.7^b^	57.8^c^	84.1^a^	58.1^c^	3.33	<.001	0.011	0.022
Proline	89.6	72.2	80.0	81.8		88.1	71.9	91.0	72.6	2.54	<.001	0.072	0.264
Serine	89.6	72.6	78.5	83.8		86.6	70.4	92.7	74.9	3.06	<.001	<.001	0.472
Tyrosine	92.3	81.7	85.4	88.6		90.0	80.8	94.6	82.7	2.22	<.001	0.001	0.126
Total amino acid	90.6	75.0	81.5	84.1		88.6	74.5	92.6	75.6	2.62	<.001	0.015	0.145

1 Number of replicates were 7 for all the simple effect groups except for the corn fines and thermoneutral temperature with 8 replicates.

2 TN = thermoneutral; HS = heat stress.

3 SD = standard deviation.

4 Temp = temperature .

## Discussion

There is a distinct difference between the nitrogen and AA content of the regular and mycotoxin-contaminated corn fine samples used in this study. Mycotoxin-contaminated corn fines had a 5% higher nitrogen concentration than regular corn, but did not translate into a superior AA profile, which was 6.8% lower than in the regular corn. This is consistent with the findings of Paczosa et al. (2025), who used a similar screening method. The discrepancy between the higher nitrogen and lower TAA in mycotoxin-contaminated corn fines suggests that a significant portion of the nitrogen may be associated with non-protein nitrogen, which is nitrogen that is not associated with real protein. Mohapatra et al. (2017) reported that fungal metabolism of carbohydrates and lipids reduces grain kernel mass, thereby increasing protein concentration. Since the corn fines are contaminated with mycotoxins, it is likely that fungi producing them consume the available energy sources in the kernel for growth, while the kernel loses mass, and the remaining nitrogen becomes more concentrated, even though the total protein quality is not improved. Furthermore, the fungal cell walls are rich in chitin, a nitrogenous polysaccharide, which could have contributed to total nitrogen in the analyzed values but does not yield nutritional AA (Brain et al., 2025; Lenardon et al., 2010). Consequently, the increased nitrogen observed in the mycotoxin-contaminated corn fines likely reflects a combination of concentrated corn proteins and indigestible fungal nitrogen, rather than an increase in high-quality dietary protein. In cereal grains, nitrogen is distributed across the endosperm, germ, and bran as protein (Shukla and Cheryan, 2001). However, a significant amount (75%) of protein is found in the endosperm, while the proteins with the best AA profile are derived from the germ (Naves et al., 2011). The pericarp, also known as the hull or bran, of the corn kernel is higher in fiber but lower in EAAs compared to the endosperm and germ (Naves et al., 2011). Corn fines are derived from the screening of corn kernels and are composed of broken kernels and hull fragments. The higher nitrogen but lower AA concentrations of the mycotoxin-contaminated corn fines used in this study may indicate its enrichment in pericarp fractions, which are typically richer in nitrogenous compounds that are not incorporated into protein.

The reduction in almost all EAAs, especially lysine and methionine, in mycotoxin-contaminated corn fines is particularly concerning and poses a challenge for poultry nutrition. Methionine and lysine are the most limiting AAs in corn-SBM based diets and are the primary drivers of feathering and growth in birds (Fernandez et al., 1994; Nukreaw and Bunchasak, 2015). The 21.6% reduction in methionine in the mycotoxin-contaminated corn fines used in this study suggests that diets incorporating high levels of corn screenings or fines would require further supplementation of synthetic DL-methionine to maintain performance. According to Pokoo-Aikins et al. (2021) and Songuine et al. (2025), broilers fed low-quality protein diets require methionine supplementation for optimum performance. Interestingly, aspartic acid and glycine were the only AAs higher in mycotoxin-contaminated corn fines than in the regular corn. According to Ospina-Rojas et al. (2012), glycine is the first-limiting NEAA in broiler chickens’ diet. Glycine is critical for protein synthesis, nitrogen retention, and uric acid formation, supporting birds' performance, collagen formation, and feather development (Siegert and Rodehutscord, 2019). The high glycine level found in the mycotoxin-contaminated corn fines may indicate its greater presence in the pericarp, supporting the report by Hood et al. (1988), who stated that the maize pericarp cell wall is abundant in glycine. These results demonstrate that the fragmentation process that creates corn fines significantly alters nutrient density. According to Senarathna et al. (2024), protein quality varies across corn fractions, with the endosperm and hull segment contributing significantly less digestible lysine than the germ. This suggests that utilizing corn fines in broiler rations without adjusting the matrix values downward for TAA will result in a diet deficiency in several limiting AAs, potentially leading to poor performance.

The use of the NFD provided a critical baseline for determining BIEAAL, supporting the methodology of Adedokun et al. (2007b) for estimating endogenous AA flow in broiler chickens. The NFD used in this study contained negligible nitrogen and TAA content, allowing for the isolation of the bird's endogenous nitrogen excretion from dietary influence. According to Alabi and Adedokun (2025), precise quantification of these basal losses is paramount when calculating SIAAD, especially when evaluating feed ingredients with different nutritional profiles or fractions in which anti-nutritional factors might vary. In this study, no specific pattern was observed in the AA profile of the semi-purified diets; however, the methionine content was reduced in the mycotoxin-contaminated corn fines diet. In poultry nutrition, diets deficient in methionine can impede growth, protein synthesis, feather and immune organ development (Conde-Aguilera et al., 2025; Liu et al., 2022). The mycotoxin-contaminated corn fines diet had a higher nitrogen concentration than the regular corn diet; however, its increase in TAA was relatively small. This discrepancy reinforced that corn fines, often composed of broken kernels and peripheral grain fractions, contain a higher proportion of NPN or structural proteins with lower AA density. Despite the significantly higher nitrogen content of the mycotoxin-contaminated corn fines diet, the increase in TAA was relatively small compared with the regular corn diet, suggesting a decrease in protein quality.

During periods of high ambient temperatures, birds reduce their FI to minimize the heat increment associated with digestion. In the present study, cyclic HS numerically decreased daily FI for birds consuming the NFD by 4.3%. Although this reduction was not statistically significant, the trend aligns with existing literature on the anorexigenic effects of HS in poultry (Brugaletta et al., 2022; Mangan and Siwek, 2024). While we had expected a significant reduction in FI, the lack of a significant difference may be due to the cyclic nature of the applied HS, which allows birds to recover during cooler nocturnal hours, thereby mitigating the sharp decline in voluntary FI often associated with chronic HS. Furthermore, the diet composition might have influenced this result, playing a protective role. According to Westerterp (2004) and Osorio et al. (2024), protein digestion and deamination yield the highest heat increment of all macronutrients. Since the NFD mainly consists of starch and fiber, with negligible protein, it generates less metabolic heat during digestion than a high-protein diet. This reduced thermogenic burden may have partially shielded the heat-stressed birds from the satiety signals usually triggered by thermal discomfort, allowing them to maintain an intake comparable to the thermoneutral group.

The findings of this study demonstrated that environmental temperature is a more potent modulator of broiler appetite than diet quality. A significant main effect of temperature resulted in a 5.5% reduction in ADFI under HS compared to TN conditions. Several authors have consistently reported a decline in broilers' FI when subjected to HS (Habashy et al., 2017; Sohail et al., 2012). This decline indicates an adaptive response in which broilers reduce their FI to lower the heat increment associated with feed digestion, thereby reducing the internal heat load that must be dissipated via other mechanisms like panting and radiation (Brugaletta et al., 2022; Olayiwola and Adedokun, 2025a; Taheri et al., 2023). The fact that diet quality did not significantly influence intake, even in the presence of contaminants, suggests that the levels of contaminants used may not have reached the threshold required to trigger organoleptic rejection or acute metabolic anorexia. While Paczosa et al. (2025), found that contaminants in corn fines triggered distinct organoleptic rejection in pigs when given a choice, the current results may be due to the fact that broilers are less sensitive to taste or have a higher tolerance threshold, thereby maintaining consumption even when substrate quality is compromised. According to Gous (2013), birds prioritize caloric intake to meet maintenance requirements. As such, the birds could have maintained their FI to meet their nutritional requirements despite dietary stressors.

The results of this study clearly demonstrate that cyclic HS aggravates the loss of endogenous nitrogen and AAs in broiler chickens. While nitrogen loss increased by approximately 31%, TAA flow rose by 36.5% under HS conditions. This significant increase suggests that the physiological challenge of high ambient temperatures induces metabolic and structural changes in the gastrointestinal tract that far exceed the baseline losses observed under TN conditions. This magnitude of loss is consistent with observations of Morales et al. (2016), who noted that HS exacerbates endogenous AA loss in growing pigs by increasing enterocyte death. This increase in endogenous nutrient flow is likely due to increased intestinal cell turnover and mucus production. Under HS conditions, birds dissipate excess heat by diverting blood flow to their periphery instead of internal organs, which reduces oxygen and nutrient delivery to enterocytes and potentially causes death (Morales et al., 2023; Olayiwola and Adedokun, 2025a). Furthermore, HS is known to induce oxidative stress and leaky gut. Varasteh et al. (2015) identified that HS actively downregulates tight junction proteins (Occludin and Zonula Occludens-1), compromising the intestinal barrier. The breakdown of tight junction proteins facilitates sloughing of enterocytes into the intestinal lumen (Dokladny et al., 2016; Lian et al., 2020). Although examination of the intestinal morphology was beyond the scope of this study, the heat-stressed birds may have experienced leaky gut, which could have led to nutrient loss.

Among the EAAs, threonine exhibited the highest absolute loss under HS. Threonine is a primary component of mucin proteins (MUC-2), which protect the intestinal epithelium (Puiman et al., 2011; Tang et al., 2021). An increase in threonine loss is often a hallmark of increased mucogenesis as the bird attempts to protect the gut lining from thermal-induced inflammation or altered microbial activity (Adedokun et al., 2011). Furthermore, the near-doubling of lysine losses is concerning, as its elevated endogenous flow may reflect increased epithelial cell desquamation or impaired reabsorption of endogenous proteins by stressed enterocytes. The NEAA profile was dominated by significant increases in glutamic and aspartic acid. Since glutamic acid is a main energy source for enterocytes and a precursor for glutathione synthesis (Blachier et al., 2009), its elevation in ileal digesta during HS could suggest rapid cell turnover or a metabolic shift to combat oxidative damage in the gut mucosa.

The effects of corn quality on nitrogen and AA digestibility indicate that diets formulated with regular corn have higher digestibility than those formulated with mycotoxin-contaminated corn fines. This significant reduction in AID of mycotoxin-contaminated corn fines likely arose from the disproportionate presence of hull fragments and broken kernel dust. Such fractions are typically higher in non-starch polysaccharides and phytate, both of which independently impair AA digestibility in broilers. Specifically, arabinoxylan, a soluble NSP, has been shown to increase endogenous AA losses and decrease total AA digestibility in chickens (Angkanaporn et al., 1994). Furthermore, dietary phytate has been reported to increase endogenous AA flow at the terminal ileum by up to 87%, depending on the level of inclusion (Cowieson and Ravindran, 2007). Collectively, Jahanian and Rasouli (2016) reported that ANFs reduced the apparent AA digestibility in diets by stimulating the secretion of digestive enzymes and increasing the sloughing of epithelial cells and mucin production. As reviewed by Grenier and Applegate (2013), mycotoxins such as fumonisins and deoxynivalenol directly target the intestinal epithelium, causing villus atrophy and reducing the expression of apical nutrient transporters. This reduction in absorptive surface area and transporter limits AA uptake. While environmental temperature alone did not significantly affect digestibility for the regular corn diet, an interaction between diet and temperature was observed for lysine, methionine, arginine, and valine. Significant interactions were also noted for aspartic acid and glycine, with the combination of HS and corn fines yielding the lowest digestibility coefficients. These AAs are highly prevalent in mucin and endogenous proteins. This aligns with the report of Ravindran (2016, 2021), who characterized aspartic acid, glycine, threonine, glutamic acid, serine, and proline as the major components of the ileal endogenous protein for pigs and chickens. The reduction in AID under HS and poor dietary quality further supports that high temperature increases the secretion of endogenous material, which then masks the true digestibility of dietary protein.

The results from this study indicate that corn quality is a critical factor influencing nutrient availability in broiler diets, with regular corn consistently demonstrating superior SIAAD compared to corn fines. The average SIAAD for TAA was 17.2% deficit in corn fines, and this could likely result from the mycotoxins, fiber, and phytate present in broken kernels and hull fragments (Adedokun et al., 2011). These components act as ANFs, increasing digesta passage rate and physically hindering the access of endogenous proteases to the protein matrix. The higher nitrogen content in fines did not translate into higher SIAAD, reinforcing that CP is a poor predictor of protein quality in feed or feed ingredients. A significant main effect of temperature was observed, with heat-stressed birds exhibiting higher SIAAD values than those in the thermoneutral environment for most AAs. While this may seem counterintuitive in association with HS, the observation may be a direct mathematical consequence of the SIAAD calculation method defined by Lemme et al. (2004). Standardized digestibility is derived by correcting apparent values for basal endogenous losses. However, it is important to recognize that the endogenous losses measured from HS birds fed the NFD in this study can no longer be considered truly 'basal' in the classical sense, as they reflect stress-elevated losses driven by increased intestinal cell turnover and mucus production under thermal challenge rather than the minimum unavoidable losses of an unstressed bird. Since the HS birds in the current study exhibited significantly elevated endogenous nitrogen flow, the correction factor applied to the HS group was disproportionately large, thereby inflating the final SIAAD coefficients relative to the TN group, suggesting that the conventional SIAAD standardization procedure may not accurately represent true amino acid digestibility under physiologically stressful conditions where endogenous losses exceed basal levels.

## Conclusion

This study demonstrates that cyclic HS and mycotoxin-contaminated corn fines both independently and in combination, reduced AA digestibility in broiler chickens. Consistent with our hypothesis, cyclic HS increased BIEAAL by 36.5% for total amino acids, whereas mycotoxin-contaminated corn fines exhibited lower protein quality and consistently lower apparent and standardized ileal AA digestibility regardless of environmental temperatures. Notably, SIAAD coefficients generated under TN conditions may not be directly additive under HS, as IEAAL measured under HS exceeded those under TN conditions. Consequently, applying SIAAD values derived from TN conditions to heat-stressed birds underestimates true AA digestibility because it does not account for the higher endogenous losses associated with HS. From a practical perspective, formulating broiler diets using SIAAD matrices without accounting for environmental stress may result in AA inadequacy when low-quality corn fractions are used. Therefore, future studies should establish environment-specific correction factors for endogenous losses to allow accurate application of SIAAD values under HS conditions. Such data would enable nutritionists to formulate more precisely for heat-stressed birds and maintain performance under commercial conditions.

## Author Contribution

**Alabi, T. J.:** Formal analysis, Investigation, Methodology, Visualization, Writing – original draft, Writing – review & editing

**Olayiwola, S. F.:** Formal analysis, Investigation, Methodology, Visualization, Writing – original draft, Writing – review & editing

**Lindeman, M. D.:** Investigation, Methodology, Writing – review & editing

**Pescatore, A. J.:** Investigation, Methodology, Writing – review & editing

**Jacob, J. P.:** Methodology, Writing – review & editing

**Ford, M. J.:** Methodology, Writing – review & editing

**Adedokun, S. A.:** Conceptualization, Formal analysis, Investigation, Methodology, Visualization, Writing – review & editing

## Disclosures

The authors declare no conflicts of interest.
